# Addition to “Independent at-Home Chemistry Project for a High School Student: Osmosis Experiments Using a U‑Tube Apparatus”

**DOI:** 10.1021/acs.jchemed.6c00526

**Published:** 2026-04-29

**Authors:** Elizabeth D. Mui, Esther Lan

U.S. Patent 12,564,813, entitled Osmosis Kit with U-Tube Apparatus and Methods of Using Thereof, issued March 3, 2026, is added to the original published work.

## Supplementary Material



## Notes

U.S. Patent 12,564,813 (PDF)

