# Supplementary material for: Addition to “Independent at-Home Chemistry Project for a High School Student: Osmosis Experiments Using a U‑Tube Apparatus”
Source: J Chem Educ. 2026 Apr 29;103(5):2810. doi: 10.1021/acs.jchemed.6c00526 (PMC13173526; doi:10.1021/acs.jchemed.6c00526)
Supplement: Supplementary file 1 [file ed6c00526_si_001.pdf]

US012564813B2

(12) United States Patent
Lan

(10) Patent No.: US 12,564,813 B2
(45) Date of Patent: Mar. 3, 2026

(54) OSMOSIS KIT WITH U-TUBE APPARATUS AND METHODS OF USING THEREOF

(71) Applicant: **Esther Lan**, Rancho Palos Verdes, CA (US)

(72) Inventor: **Esther Lan**, Rancho Palos Verdes, CA (US)

(*) Notice: Subject to any disclaimer, the term of this patent is extended or adjusted under 35 U.S.C. 154(b) by 817 days.

(21) Appl. No.: **17/827,930**

(22) Filed: **May 30, 2022**

(65) **Prior Publication Data**

US 2023/0191327 A1 Jun. 22, 2023

Related U.S. Application Data

(63) Continuation-in-part of application No. 17/554,602, filed on Dec. 17, 2021, now abandoned.

(51) **Int. Cl.**

C02F 1/44	(2023.01)
B01D 61/00	(2006.01)
G09B 23/12	(2006.01)
C02F 101/34	(2006.01)

(52) **U.S. Cl.**

CPC ***B01D 61/002*** (2013.01); ***C02F 1/445*** (2013.01); ***G09B 23/12*** (2013.01); *C02F 2101/34* (2013.01)

(58) **Field of Classification Search**

CPC C02F 1/445; C02F 2103/08; C02F 1/44; C02F 2101/34; B01D 61/005; B01D 61/002; B01D 2313/205; B01D 61/0022; B01D 63/087; G09B 23/12

See application file for complete search history.

(56) **References Cited**

U.S. PATENT DOCUMENTS

4,475,556	A	10/1984	Reiff
7,476,103	B1	1/2009	Norman
8,888,145	B1	11/2014	Crompton
10,195,536	B2	2/2019	Visser
2018/0280881	A1	10/2018	Trones

FOREIGN PATENT DOCUMENTS

CN 213446510 U * 6/2021

OTHER PUBLICATIONS

AIP Conf. Proc. 2031, 020020 (2018). (Year: 2018).*

(Continued)

Primary Examiner — Pranav N Patel

(57) **ABSTRACT**

A complete kit, including components to assemble a U-tube apparatus, materials, and methods of using thereof, for the purpose of osmosis pedagogy, is described. The self-contained kit makes possible hands-on osmosis experiments that may be conducted safely at home, and is well-suited for STEM (science, technology, engineering and mathematics) education. The kit supplies unmodified glycerin and unmodified dextran as solutes, and components to assemble a watertight U-tube apparatus capable of accurate, repeatable quantitative measurements and bidirectional osmosis. One method describes osmotic rate measurements using glycerin as solute under varying parameters of solute concentration, temperature, and osmosis direction. Another method describes the comparison of experimental measurement of osmotic pressure at equilibrium to theoretical prediction using dextran as solute.

1 Claim, 12 Drawing Sheets

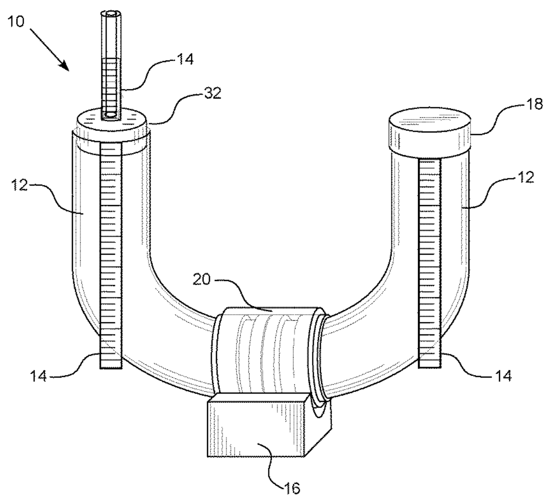

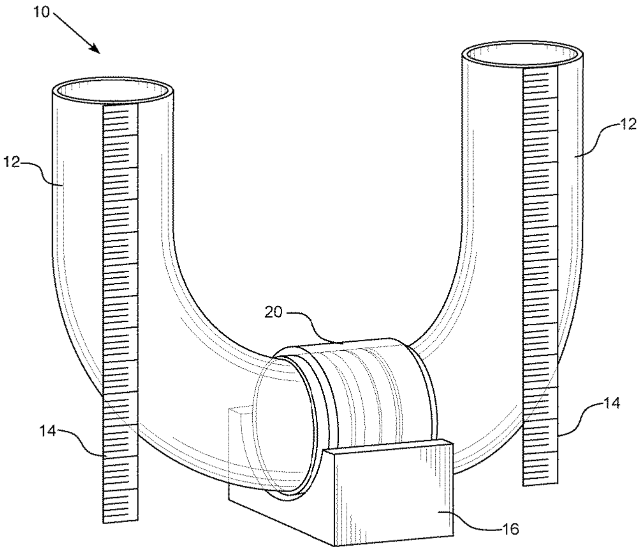

(56) **References Cited**

OTHER PUBLICATIONS

Desalination and Water Treatment 57 (2016) 13425-13432. (Year: 2016).*
English language machine translation of CN213446510-U, 5 pages, No Date.*
PW. Rodriguez-Duenas, H. A. Bernal Munoz, D. C. Rodriguez Burbano, "A hands-on activity to teach the osmosis phenomenon," Advances in Physiology Education, 2022, pp. 251-258, vol. 46, issue 2. https://doi.org/10.1152/advan.00186.2020.
E. Bernacka, H. Jaroszek, M. Turek, p. Dydo, D. Czechowicz, K. Mitko, Application of Waste Glycerol as a Draw Solution for Forward Osmosis, Membranes, 2021, pp. 44-57, vol. 12, issue 1. https://doi.org/10.3390/membranes12010044.
W. Fang, C. Liu, L. Shi, R. Wang, Composite forward osmosis hollow fiber membranes: Integration of RO- and NF-like selective layers for enhanced organic fouling resistance, Journal of Membrane Science, 2015, pp. 147-155, vol. 492, issue 4. https://doi.org/10.1016/j.memsci.2015.05.045.
S. Akca, p. Arpacay, N. Mcevoy, O. Prymak, W. J. Blau, M. Ulbricht, "Feasibility of graphene-polymer composite membranes for forward osmosis applications," Materials Advances, 2021, pp. 6439-6454, vol. 2., issue 19. https://doi.org/10.1039/D1MA00424G.
C. Ding, X. Zhang, L. Shen, J. Huang, A. Lu, F. Zhong, Y. Wang, "Application of polysaccharide derivatives as novel draw solutes in forward osmosis for desalination and protein concentration," Chemical Engineering Research and Design, 2019, pp. 211-220, vol. 146. https://:10.1016/j.cherd.2019.04.005.
S. W. Lim, S.-K. Mah, Z. H. Lee, S.-P. Chai, "A study of water permeation using glycerol as the draw solution with thin film composite membranes in forward osmosis and pressure retarded osmosis configurations," AIP Conference Proceedings, 2018, pp. 020020-1 to 020020-5, vol. 2031, issue 1. https://doi.org/10.1063/1.5066976.
Instruction manual for Neo-Sci osmosis simulation activity model 30-1125 (this apparatus is not watertight over the longterm, has been discontinued).
Instruction manual for Diffusion-Osmosis Apparatus ME-6940.

* cited by examiner

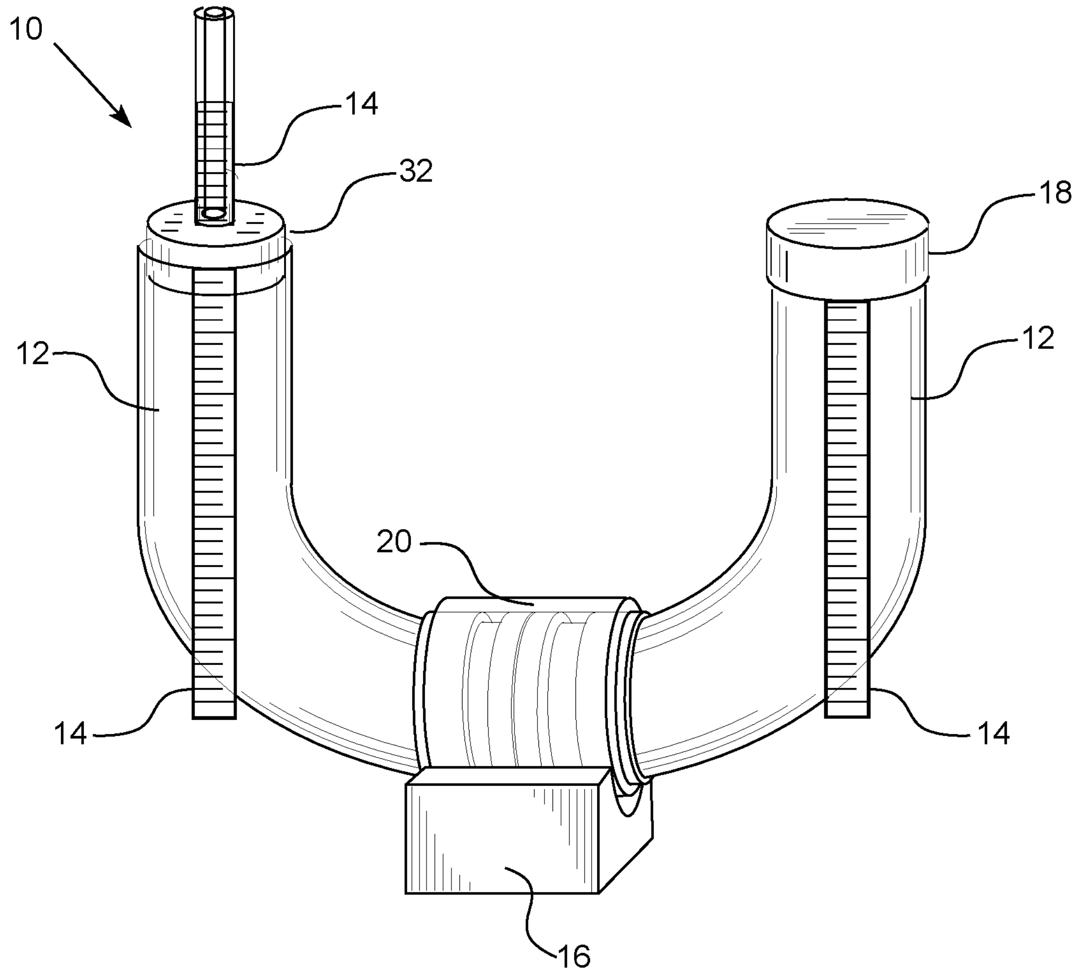

FIG. 1A

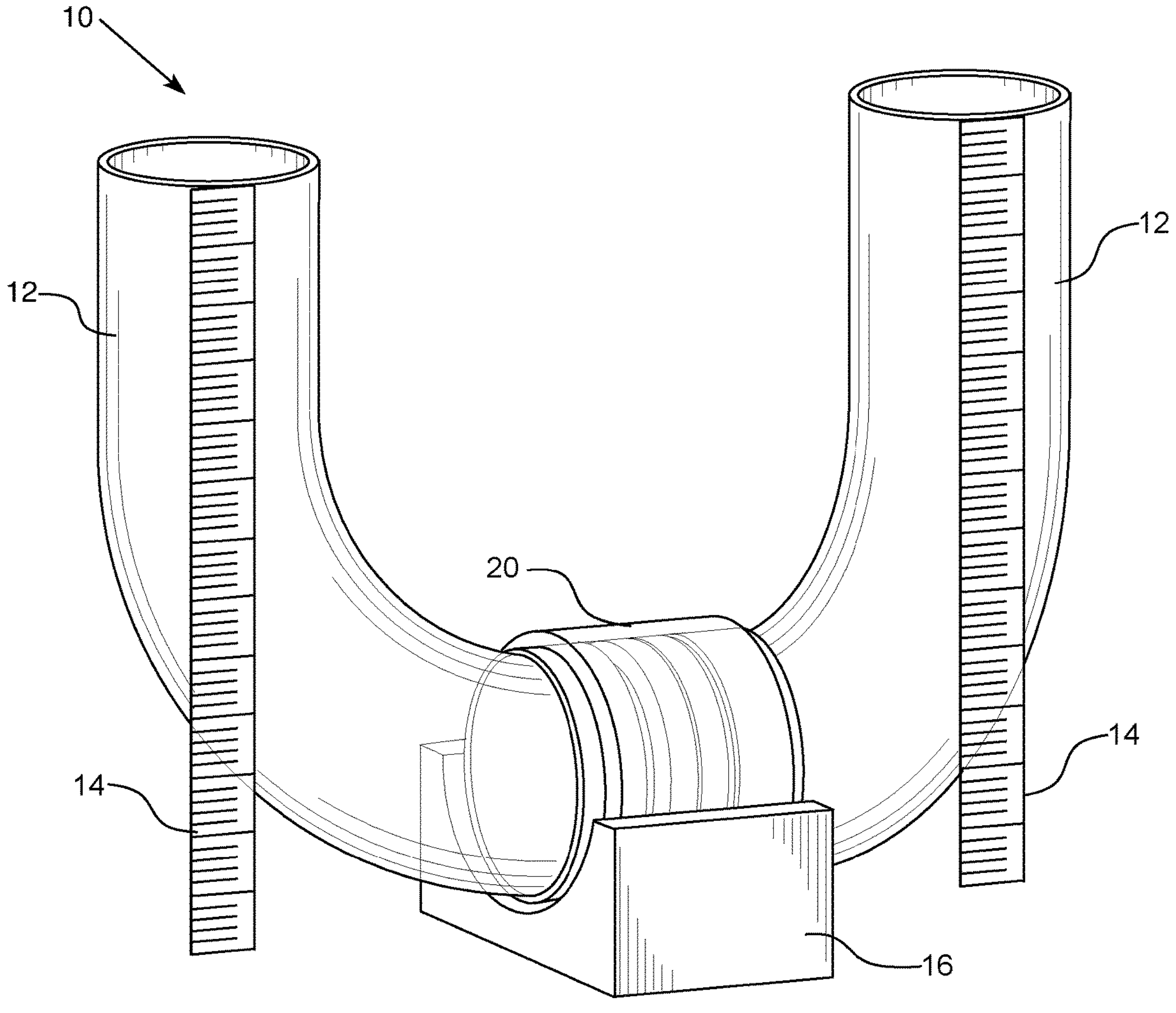

FIG. 1B

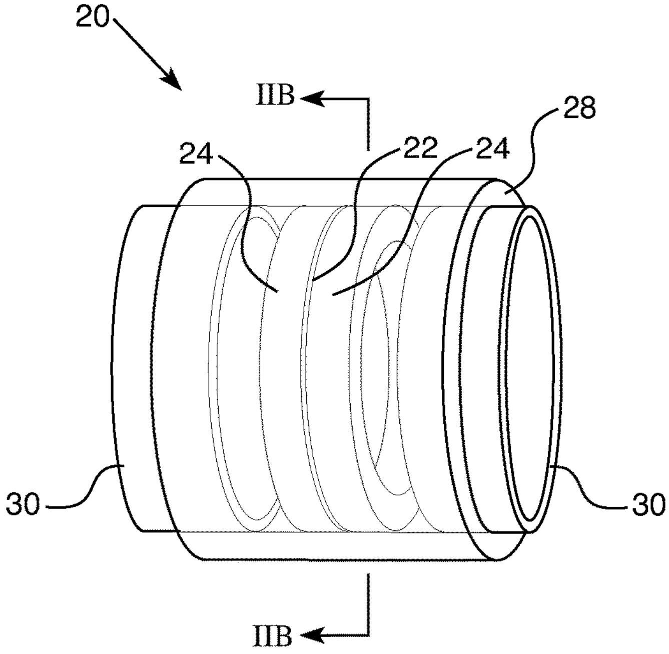

FIG. 2A

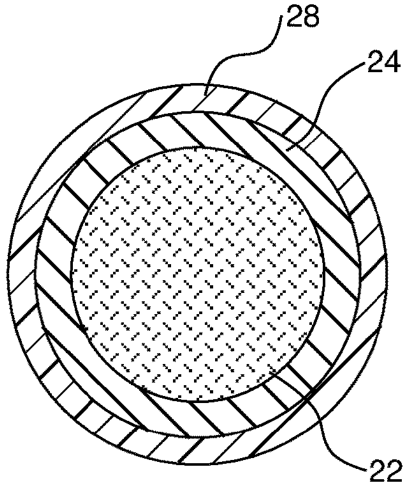

FIG. 2B

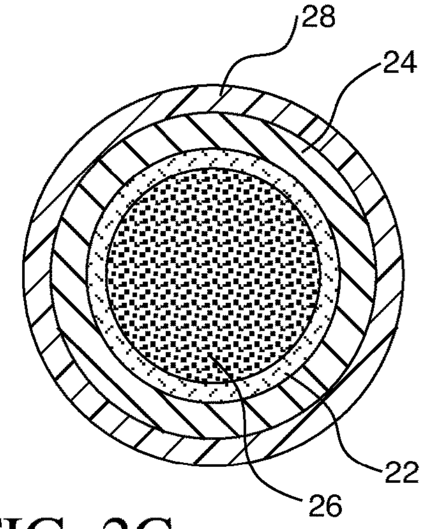

FIG. 2C

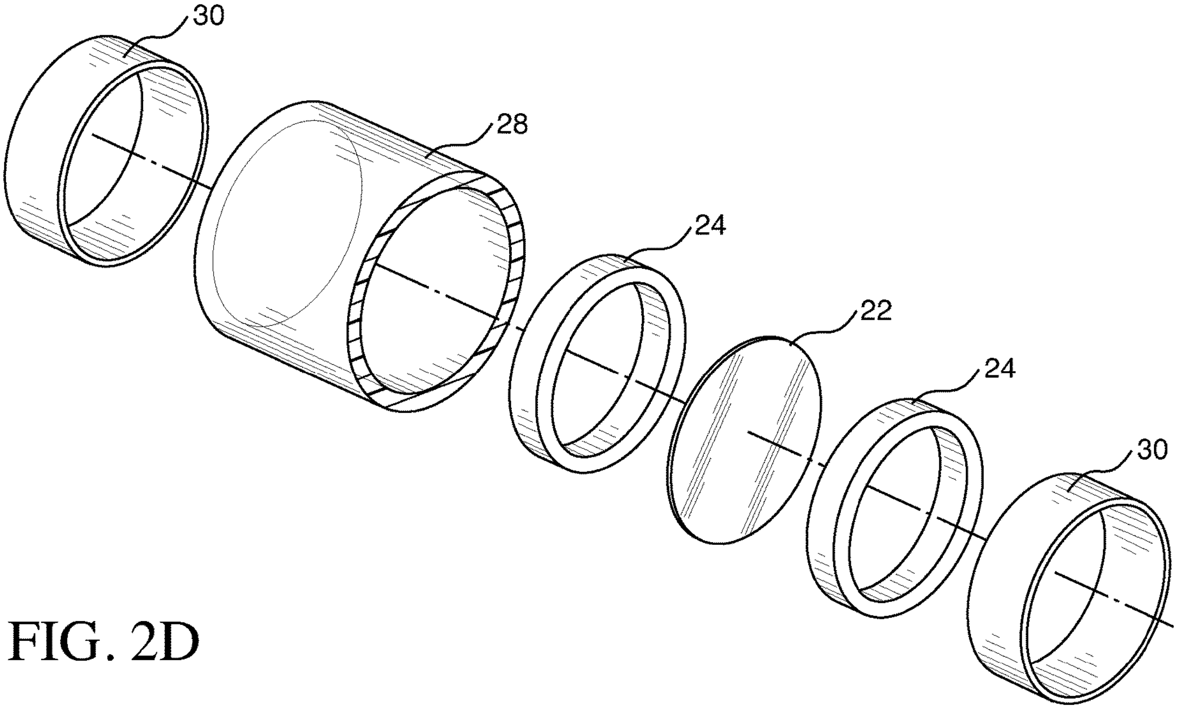

FIG. 2D

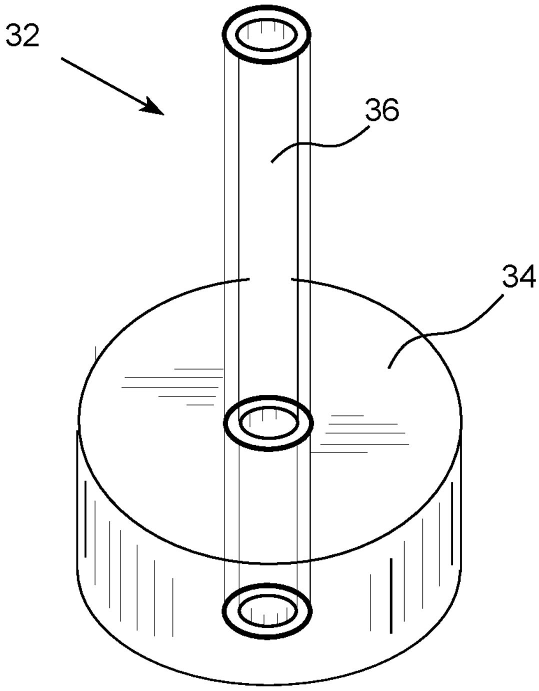

FIG. 3A

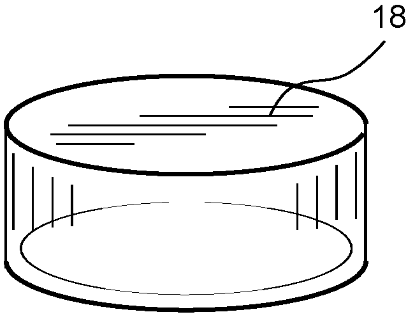

FIG. 3B

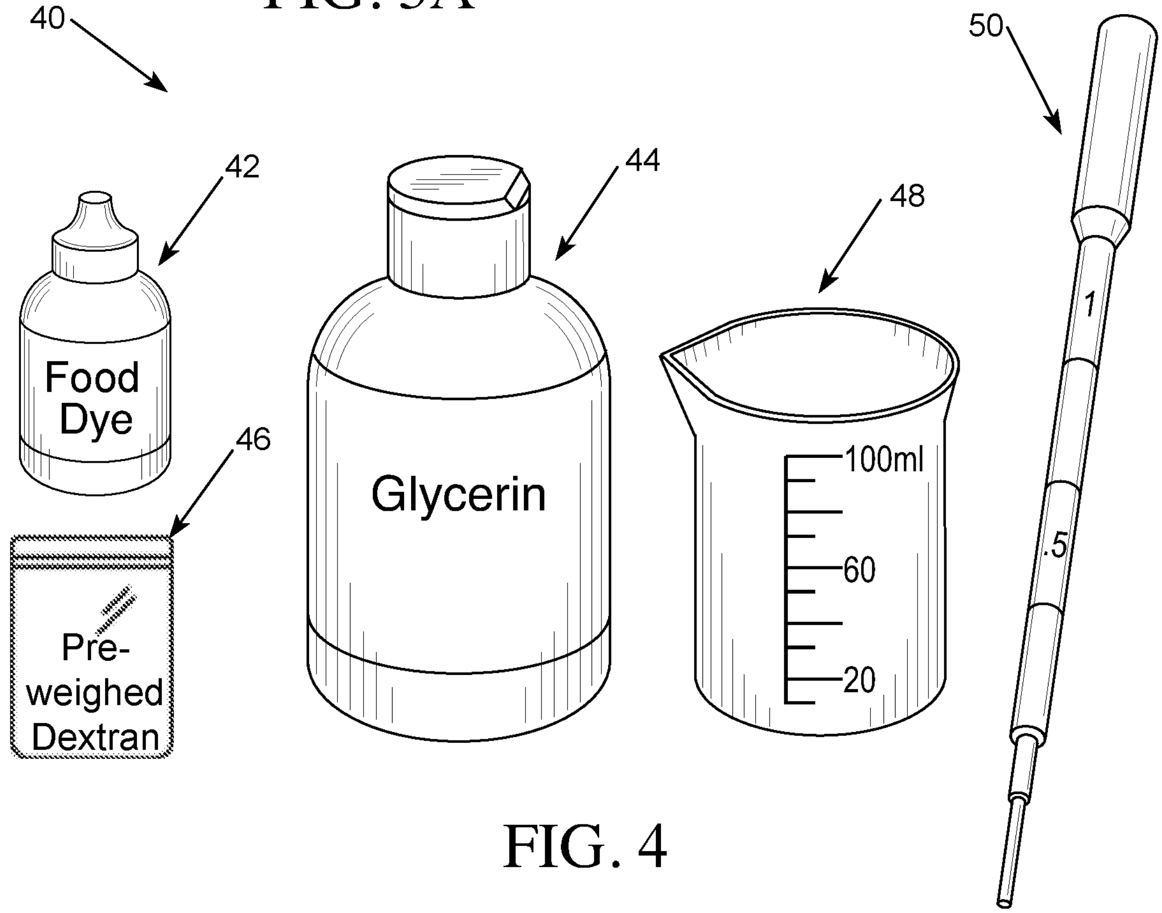

FIG. 4

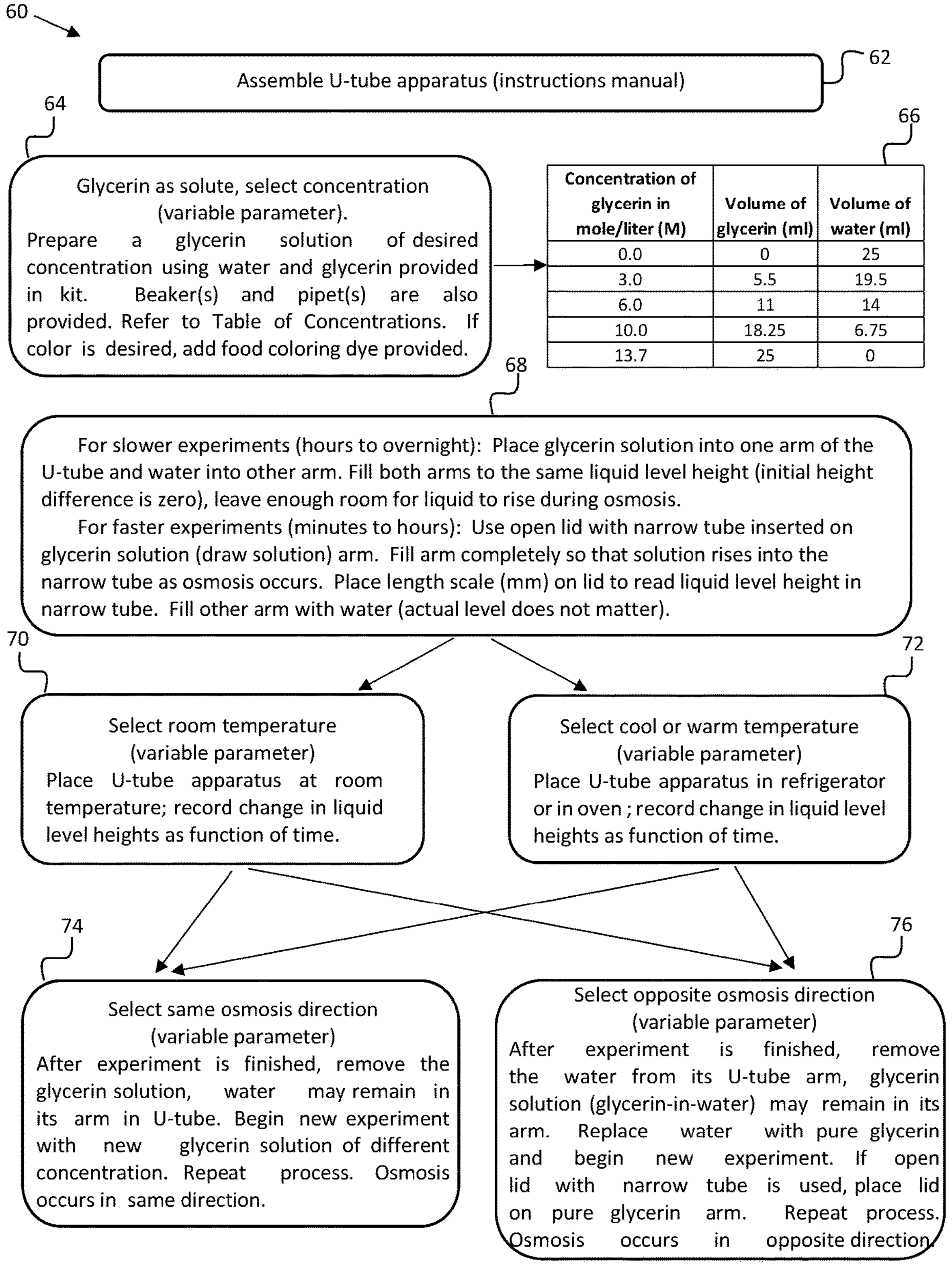

Concentration of glycerin in mole/liter (M)	Volume of glycerin (ml)	Volume of water (ml)
0.0	0	25
3.0	5.5	19.5
6.0	11	14
10.0	18.25	6.75
13.7	25	0

FIG. 5

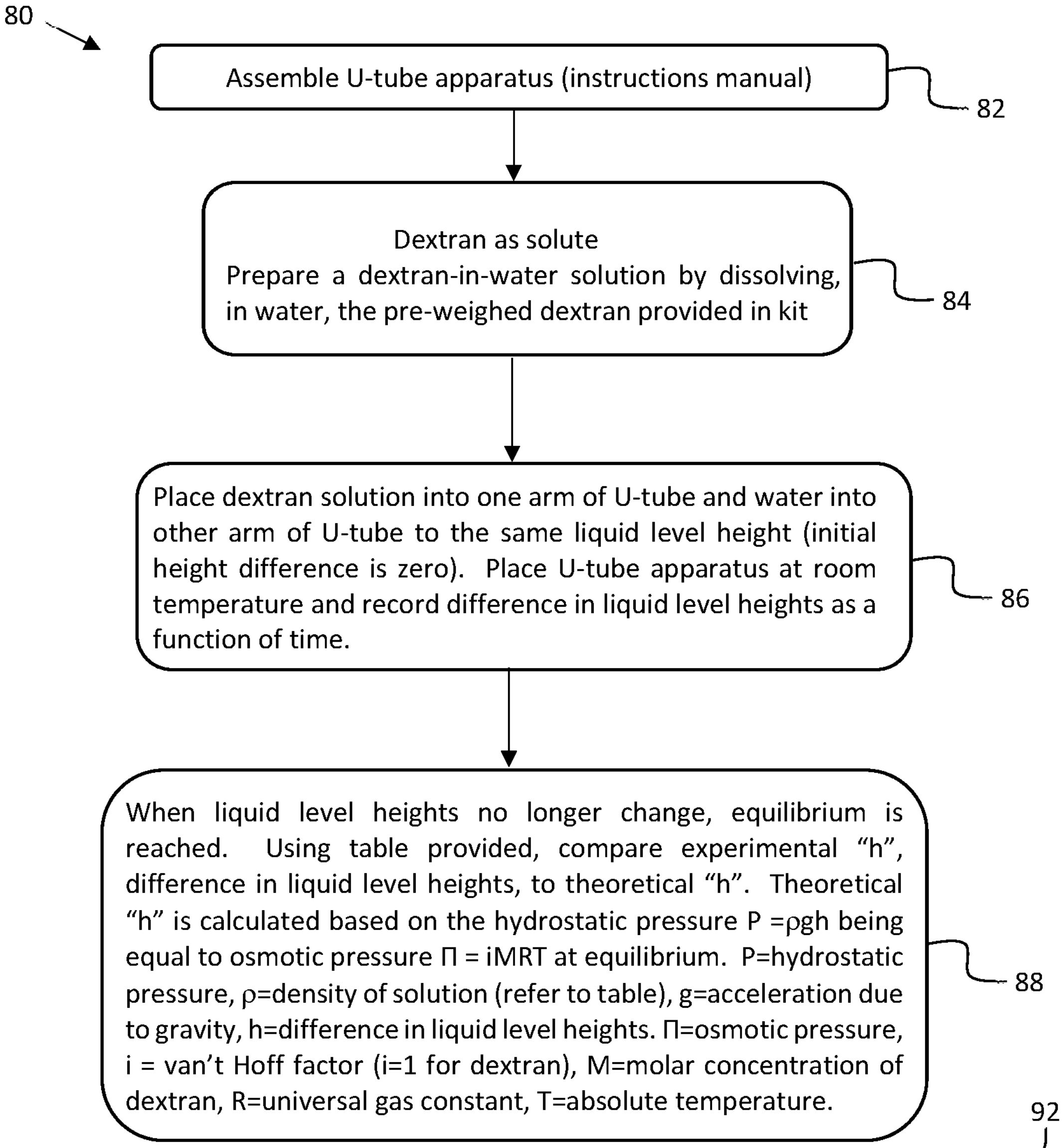

90

Table of dextran solution density, assuming dextran of average molecular weight M_w~500,000 g/mole

Molarity of dextran (mM)	Density (kg/m^3)
0.0 (pure water)	998
0.1	1018
0.2	1035
0.3	1055
0.4	1080

92

Table of calculated "h" (difference in liquid level heights at equilibrium), using equation iMRT = ρgh

Molarity of solute (mM) (i=1 for non-electrolyte such as dextran)	Calculated "h" (difference in liquid level heights, mm)
0.06	14.9
0.08	19.9
0.10	24.8
0.12	29.8
0.14	34.8
0.16	39.7
0.18	44.7
0.20	49.7

FIG. 6

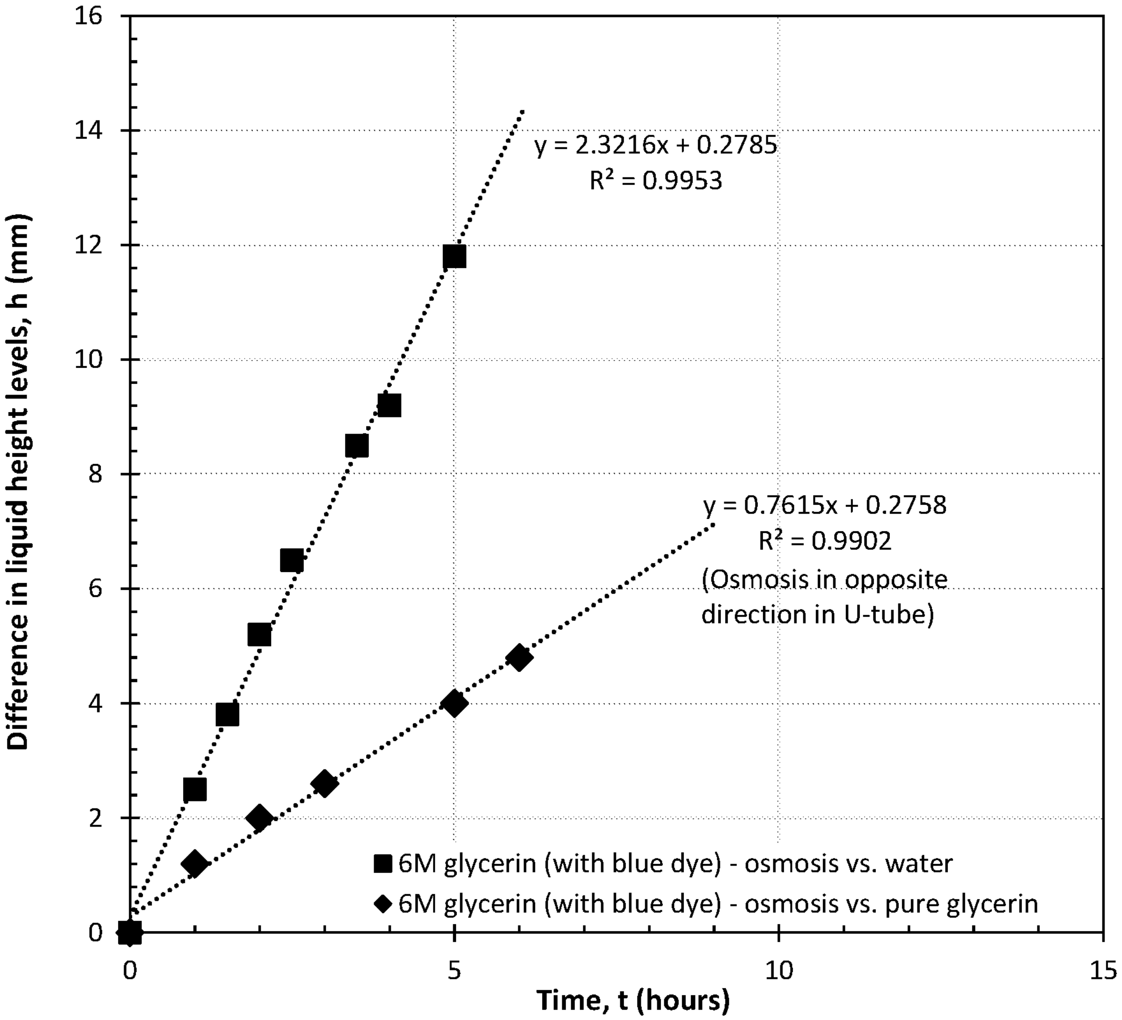

16
14
12
10
8
6
4
2
0
Difference in liquid height levels, h (mm)
y = 2.3216x + 0.2785
R² = 0.9953
y = 0.7615x + 0.2758
R² = 0.9902
(Osmosis in opposite direction in U-tube)
6M glycerin (with blue dye) - osmosis vs. water
6M glycerin (with blue dye) - osmosis vs. pure glycerin
0
5
10
15
Time, t (hours)

FIG. 7

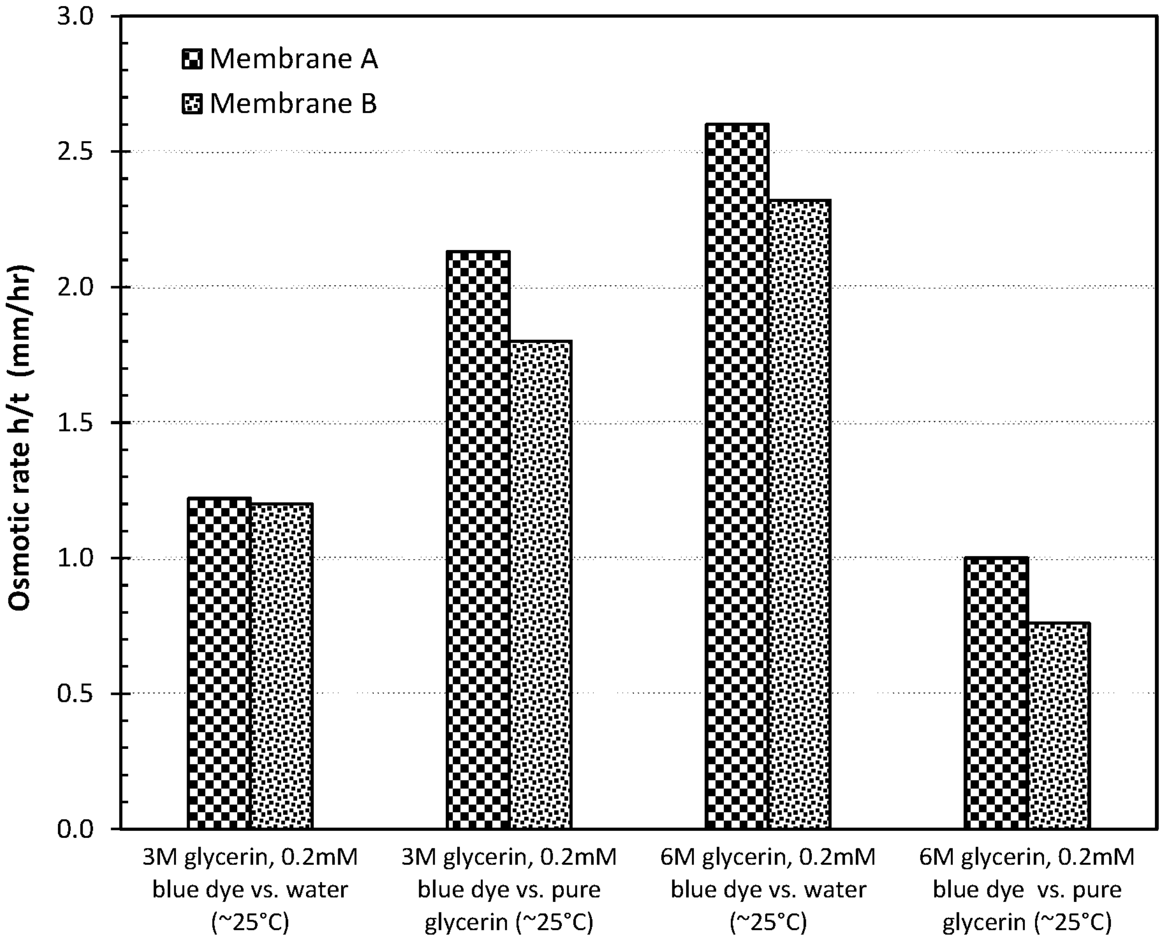

Osmosis occurred in opposite directions in U-tube depending on whether pure glycerin or water was in the other U-tube arm.

FIG. 8

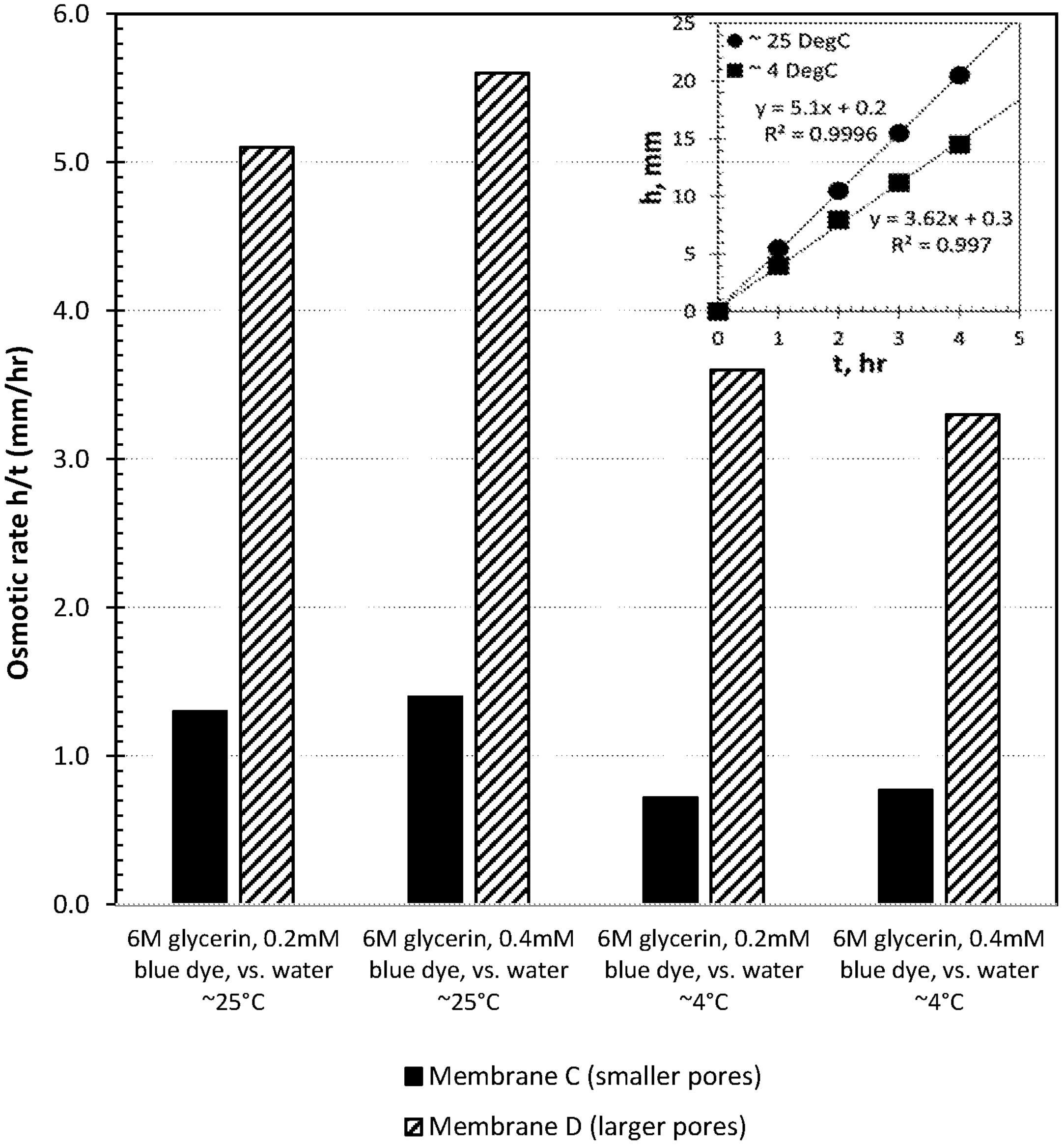
Osmotic rate h/t (mm/hr)
6.0
5.0
4.0
3.0
2.0
1.0
0.0
6M glycerin, 0.2mM blue dye, vs. water ~25°C
6M glycerin, 0.4mM blue dye, vs. water ~25°C
6M glycerin, 0.2mM blue dye, vs. water ~4°C
6M glycerin, 0.4mM blue dye, vs. water ~4°C
Membrane C (smaller pores)
Membrane D (larger pores)
~ 25 DegC
~ 4 DegC
y = 5.1x + 0.2
R² = 0.9996
y = 3.62x + 0.3
R² = 0.997
h, mm
t, hr
0
5
10
15
20
25
0
1
2
3
4
5

FIG. 9

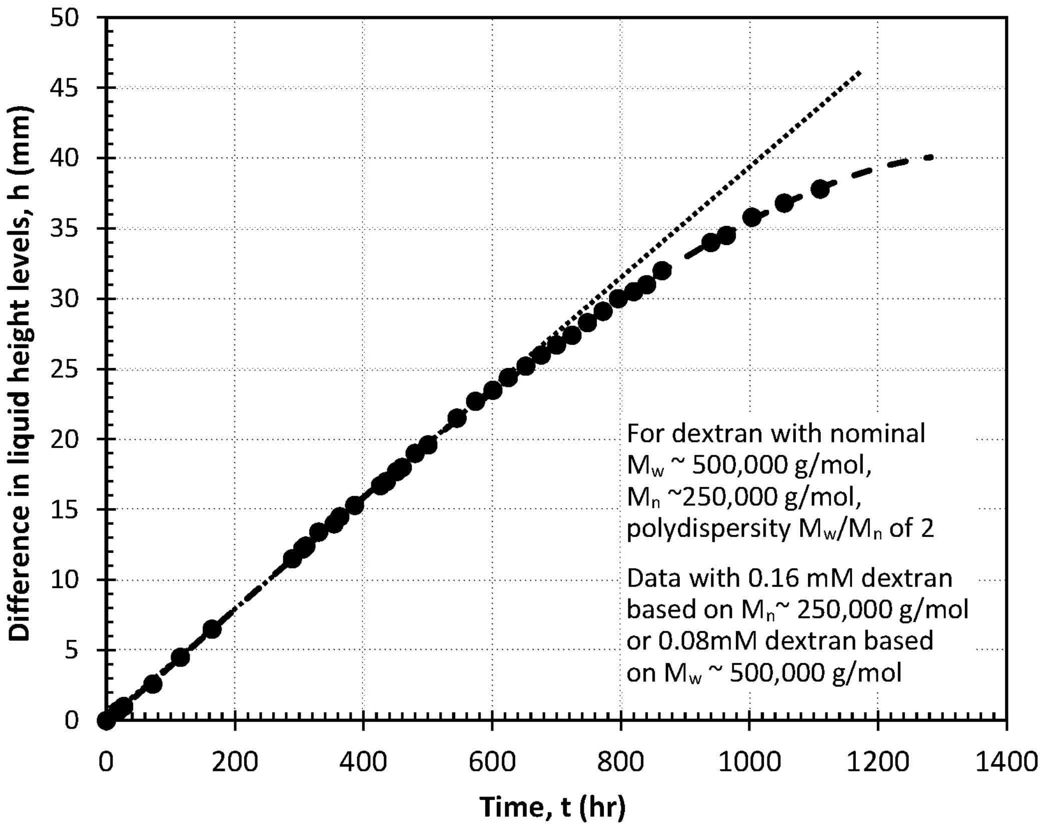

50
45
40
35
30
25
20
15
10
5
0
Difference in liquid height levels, h (mm)
0
200
400
600
800
1000
1200
1400
Time, t (hr)
For dextran with nominal
M_w ~ 500,000 g/mol,
M_n ~250,000 g/mol,
polydispersity M_w/M_n of 2
Data with 0.16 mM dextran
based on M_n~ 250,000 g/mol
or 0.08mM dextran based
on M_w ~ 500,000 g/mol

FIG. 10

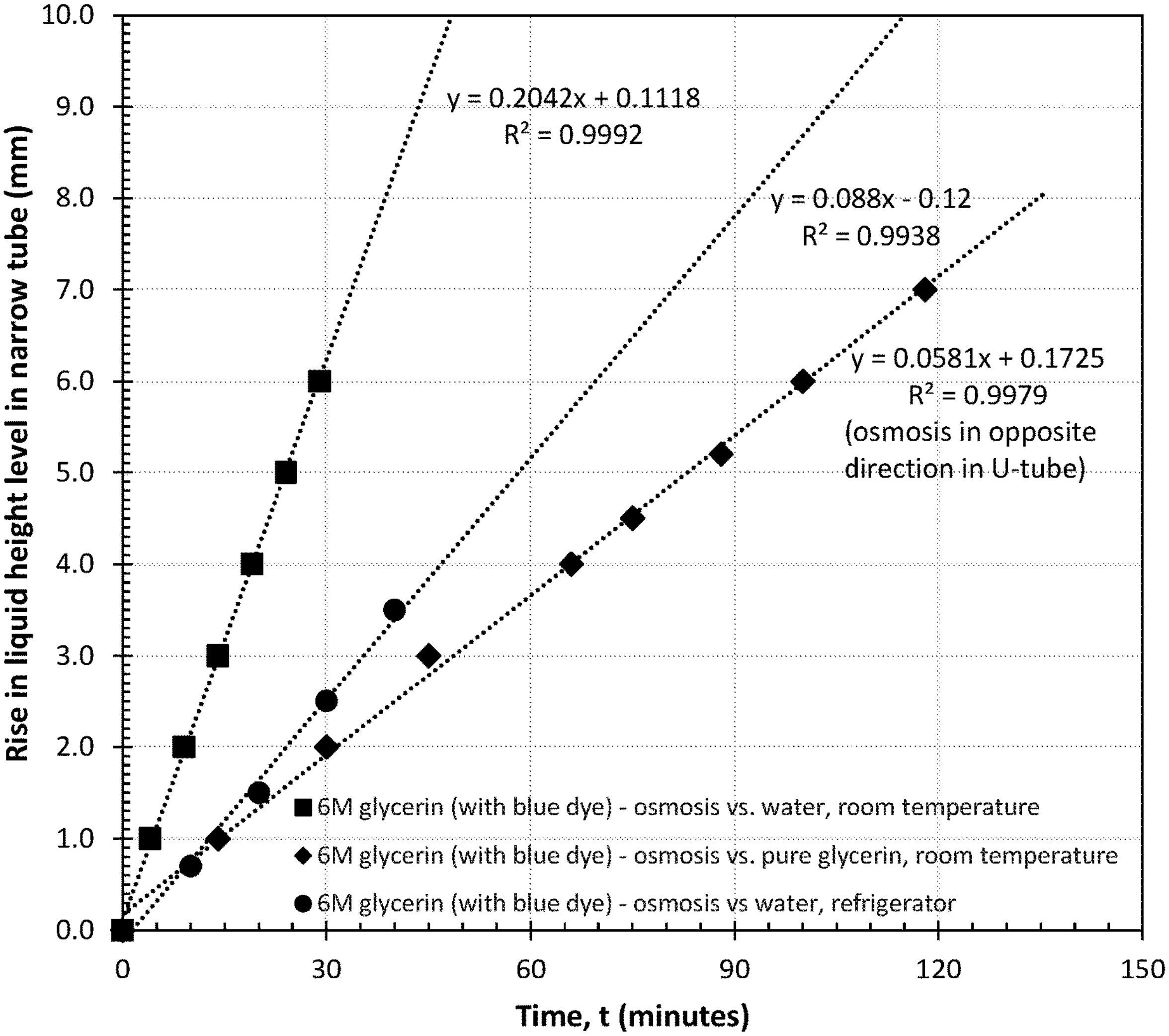

y = 0.2042x + 0.1118
R^2 = 0.9992
y = 0.088x - 0.12
R^2 = 0.9938
y = 0.0581x + 0.1725
R^2 = 0.9979
(osmosis in opposite direction in U-tube)
6M glycerin (with blue dye) - osmosis vs. water, room temperature
6M glycerin (with blue dye) - osmosis vs. pure glycerin, room temperature
6M glycerin (with blue dye) - osmosis vs water, refrigerator
Rise in liquid height level in narrow tube (mm)
Time, t (minutes)
0.0
1.0
2.0
3.0
4.0
5.0
6.0
7.0
8.0
9.0
10.0
0
30
60
90
120
150

FIG. 11

OSMOSIS KIT WITH U-TUBE APPARATUS AND METHODS OF USING THEREOF

CROSS-REFERENCE TO RELATED APPLICATION

This non-provisional application is a continuation-in-part of a non-provisional application entitled "Osmosis Kit with U-tube Apparatus and Methods of Using Thereof", U.S. Ser. No. 17/554,602, filed Dec. 17, 2021, which is incorporated by reference herein in its entirety. For the purpose of simplicity, the present disclosure will only reference the elements of the non-provisional application U.S. Ser. No. 17/554,602 that are relevant to the innovations disclosed in this application.

TECHNICAL FIELD OF THE INVENTION

The present invention pertains generally to a complete educational kit which includes components to assemble a watertight U-tube apparatus, necessary chemicals, and methods of using the kit for a variety of osmosis experiments. The self-contained kit does not require external tools nor electrical power and is particularly well-suited for osmosis pedagogy in STEM (science, technology, engineering and mathematics) education.

BACKGROUND OF THE INVENTION

The COVID-19 pandemic has created a greater need for meaningful hands-on science experiments for students that can be conducted safely at home, making possible a remote laboratory experience. Ideally, the science experiments involve quantitative measurements, not only qualitative observations, and do not require additional equipment (e.g., analytical mass balance, magnetic stir plate, heat plate, fume hood, etc.) nor hazardous waste disposal. The phenomenon of osmosis naturally lends itself to experiments that meet the criteria listed above. However, a complete self-contained kit with a suitable apparatus, accompanying chemicals and supplies, and proper methods to study osmosis that possess the features of being reusable, safe to use at home, watertight over the long-term, enables accurate measurements of liquid flow while minimizing liquid evaporation, and permits bidirectional osmosis (osmosis in two directions), has been elusive.

Osmosis is the flow of a solvent through a semipermeable membrane from a solution of lower solute concentration to a solution of higher solute concentration. An ideal semipermeable membrane allows solvent, but not solute, to pass through. In nature (e.g., plant cells and red blood cells), water is the solvent flowing during osmosis and water is the most common solvent. There are several definitions of osmotic pressure. One definition is the pressure that must be applied to the solution to stop the flow of solvent, another definition is the force driving the solvent into the solution containing higher solute concentration, and yet another is the hydrostatic pressure required to interfere with osmosis. Regardless of which definition is used, there is a pressure that drives or prevents osmosis. If osmosis occurs unimpeded, eventually, equilibrium is reached. In STEM education, a complete kit with accompanying methods that make possible at-home experiments to quantitatively measure osmosis rates under a variety of conditions and to determine osmotic pressure at equilibrium, without external tools or external equipment, is therefore desirable, but not yet available.

An osmosis U-tube apparatus consists of two arms or compartments separated by a semipermeable membrane, with one arm or compartment containing solvent, which acts as the feed solution, while the other arm or compartment contains solute solution, which acts as the draw solution. For osmosis pedagogy, water is a good solvent or feed solution. It is possible to quantitatively measure the rate at which passive forward osmosis occurs with water (feed solution) flowing from its compartment or arm, across the semipermeable membrane, into the solute solution compartment or arm (draw solution). Passive osmosis occurs spontaneously with no external pressure, no external equipment, and no energy input.

A U-tube apparatus that is watertight over the long-term requires proper design of a connection between two arms separated by a semipermeable membrane. A watertight seal in the connection using a minimum number of parts, minimal assembly, and does not require any external tools is desired. One design to achieve a watertight connection between two compartments (or arms) is to use a threaded connection (U.S. patent 2018/0280881). Threaded fittings, however, are vulnerable to damage as well as wear and tear, especially for non-metals undergoing frequent connections and disconnections, eventually causing teaks. Machining internal and external threads into materials that are brittle in nature is likely to damage the material. It is also possible to use a design with a sealing ring between compartments (or arms), but precise shape or contour of the fitting (e.g., rounded surfaces, grooves, rims, etc.) is required to accommodate a sealing ring (U.S. Pat. No. 8,888,145). Moreover, U.S. Pat. No. 8,888,145 explicitly states that an external press tool is required because manual force from an individual is not significant enough to compress, and the seal is a permanent seal, the components cannot be separated nor can sealing ring be removed after seal is formed. In a commercially available U-tube apparatus, a watertight connection is achieved using O-rings, raised pegs, bolts, and alignment tabs (PASCO Diffusion-Osmosis Apparatus ME-6940). Rodriguez-Duenas, et. (2022) reports a hands-on activity to teach osmosis, and the laboratory-made osmosis device similar to the PASCO apparatus, with the two compartments held together by an O-ring, retaining-plate, and screws.

In order to perform osmosis experiments, suitable solutes are necessary. If experiments are to be conducted at home, it is essential that any chemicals that are used are non-toxic. Sucrose (table sugar) is the most commonly used solute for educational purposes, with sucrose-in-water solutions used as draw solution. Both the PASCO apparatus manual and Rodriguez-Duenas journal article (2022) recommend sucrose as the solute, but sucrose is a solid powder that requires an analytical mass balance to measure. For the pedagogy of osmosis, liquid glycerin (also known as glycerol) is a superior solute to solid sucrose. The utilization of liquid glycerin as solute provides several benefits: 1) ease of sample preparation because volumes of glycerin and water may be measured using beaker(s) and pipet(s) without an analytical mass balance, 2) large working range in solute concentration, from 0 to 13.7 M (mol/L) for glycerin (glycerin has a molar mass of 92.1 g/mol, density of 1.26 g/cm^3, pure glycerin is 13.7 M) compared to solid sucrose which, in practice, may be prepared only up to ~2 M without a heat/stir plate, 3) ease of clean-up because glycerin, a polyol, is fully miscible with water, 4) long term storage because glycerin solutions do not undergo biofouling and have an indefinite shelf-life, in contrast to solutions with sucrose, glucose, etc. that undergo biofouling due to bacteria and

mold, 5) glycerin may be used as a humectant on membranes; it is possible to coat membranes with glycerin to prevent them from drying out during storage and reuse them, without storing membranes in water.

Glycerin or glycerol has been evaluated as a draw solution in forward osmosis with water as feed solution because of its potential use in water desalination, wastewater purification, and production of drinking water from seawater (Lim, et. al, 2018, Bernacka, et. al, 2021). Forward osmosis in these studies, however, was not completely passive. Lim, et. al. (2018) used lab bench-top equipment to pump both feed and draw solutions at constant volumetric flow rates. Bernacka, et. al, (2021) used a Sterlitech laboratory module CF042D-FO with feed flow pump to ensure the same linear flow velocity on both feed and draw sides of the membrane. Compared to sodium chloride (NaCl), glycerol was inferior (Lim, et. al, 2018), because the theoretical value of van't Hoff factor for non-ionic glycerol is 1 whereas van't Hoff factor for fully dissolved ionic Na^+ and $Cl^-$is 2, so that osmotic pressure for NaCl is theoretically double that of glycerol at the same concentration. Adding NaCl to glycerol to create a mixture improves the osmotic pressure and water flux (Bernacka, et. al., 2021).

In the present disclosure, a U-tube apparatus is described for the purpose of osmosis pedagogy, and experiments will be completely passive, with no pumps or other equipment involved. When water (solvent) is in one arm of the U-tube apparatus and a solution containing solute glycerin (e.g., glycerin-in-water solution) is in the other arm, water acts as the feed solution and the glycerin-in-water solution acts as the draw solution. However, with the same glycerin-in-water solution in its arm of the U-tube, if undiluted or pure glycerin replaces water in the other arm, the glycerin-in-water acts as the feed solution and the undiluted glycerin acts as the draw solution, because there is now higher solute concentration in the undiluted glycerin. In this configuration, water (solvent) flows from the glycerin-in-water arm across the semipermeable membrane into the undiluted glycerin arm. The feature that water may flow in two directions is defined as bidirectional osmosis. In this case, the two directions are opposite to each other in a U-tube. There have been no reports of bidirectional osmosis using glycerin, which makes this usage innovative.

Polysaccharide derivatives, such as dextran derivatized with anionic carboxymethyl (CM) groups, have also been evaluated as a draw solution in forward osmosis for desalination (Ding, et. al, 2019). The degree of substitution, which is the average number of sodium (Na) carboxymethyl (CM) groups substituted per anhydroglucose unit, was about one in all cases. Since anhydroglucose unit has molecular weight of about 160 g/mol, for CM-dextran-1000 (dextran of 1000 g/mol molecular weight), there would be about 6 Na^+ cations in addition to 6 $CH_2CO_2^-$ anions per each CM-dextran-1000 molecule. For CM-dextran-20000 (dextran of 20,000 g/mol molecular weight), there would be about 120 Na+ in addition to 120 $CH_2CO_2^-$ per each CM-dextran-20000 molecule. The CM-dextran was compared to 1M NaCl as draw solution, and due to the many ions (both cations and anions) in the CM-dextran, there was higher water flux in forward osmosis using CM-dextran compared to NaCl. In the osmosis kit in the present disclosure, dextran that is unmodified, non-derivitized, also known as native dextran, is another solute (used in draw solution) for osmosis pedagogy. Native dextran has been included in feed solutions to determine foulant resistance or foulant rejection in membranes (Akca, et. al, 2021, Fang, et. al, 2015). In these studies, the purpose was to investigate how much the membrane would be affected using native dextran as a model foulant in a feed solution.

In the present disclosure, the purpose of unmodified or native dextran as solute is to determine osmotic pressure at equilibrium, which is equal to the hydrostatic pressure at equilibrium. When equilibrium is reached, the hydrostatic pressure can be measured by the difference in liquid column heights between the two U-tube arms, one containing water (feed solution) and the other containing dextran solution (draw solution). In order to measure this hydrostatic pressure, dextran should be unmodified, non-derivitized (i.e., in its native form) so that it remains non-ionic (also referred to as neutral or a non-electrolyte), to keep osmotic pressure to a minimum. In so doing, the rise in liquid column height is kept to a reasonable height so that it can be measured in a U-tube. Moreover, native dextran may be disposed in the drain, which may not be true when the dextran has been derivatized or modified. There have been no previous reports of using native dextran for such a purpose, which makes this disclosure an innovative method.

BRIEF SUMMARY OF THE INVENTION

The present invention describes a complete self-contained kit which can be used to quantitatively measure the osmotic rate (i.e., rate of osmosis) as a function of a wide range of experimental parameters, and to determine the osmotic pressure at equilibrium by measuring the hydrostatic pressure. The osmosis kit comprises dissembled components of a U-tube apparatus, necessary chemicals and measuring tools, and an instructions manual. Methods to use the kit to determine osmotic rates, as well as osmotic pressure at equilibrium, are also disclosed.

The U-tube apparatus has two removable, optically clear curved arms that can be assembled, disassembled, and reassembled into a center mount. The architecture of the center mount makes possible a watertight U-tube, ensures its structural integrity, maintains it in upright position on a stand, and keeps the overall apparatus as small and compact as possible. The center mount contains a semipermeable membrane affixed between two washers, and the washer-membrane-washer sandwich is encased in a protective tube. There is a removable, non-rigid, and deformable sleeve on each end of the protective tube. The purpose of each sleeve is to allow one curved arm to be reversibly inserted into the center mount in a watertight manner.

The advantage of this U-tube design is that a watertight connection between two arms and the center mount containing a semipermeable membrane is achieved without over-engineering the connection, even when arms are filled with liquid and the center mount lies horizontally at the bottom of the U. Every component of the apparatus (arms, sleeves, center mount with semipermeable membrane, stand) is washable, reusable, and can be disassembled from and reassembled into the U-tube apparatus without damaging any of the components. Moreover, the apparatus minimizes the sample volume of liquid needed during osmosis experiments.

Two different types of lids for the arms are provided in the kit, an open lid with a removable narrow tube and a closed lid that caps a U-tube arm tightly. When a U-tube arm has an open lid with the narrow tube inserted, and the arm is completely filled so that as liquid height level increases during osmosis, liquid enters the narrow tube, measurements of osmotic rates are faster because liquid rises faster in a smaller diameter narrow tube compared to a larger diameter

U-tube arm. Closed lid(s) that tightly caps U-tube arm(s) is also provided if it is necessary to prevent liquid evaporation during osmosis experiments. The kit contains length scales for measurement of liquid level heights.

The chemicals provided in the osmosis kit are 1) unmodified, non-derivitized glycerin, 2) unmodified, non-derivitized, pre-weighed high molecular weight dextran, and 3) food coloring dye. For better visualization and aesthetics, food coloring dye may be added, although dye is not required for osmosis. All chemicals in the kit are water-soluble and non-toxic so that experiments may be conducted at home, with chemicals safely disposed as household waste or in the drain. For measuring and dispensing liquids, disposable graduated beaker(s) or graduated cylinder(s) and disposable pipet(s) are also provided.

Two methods of using the U-tube apparatus are disclosed as part of this invention. In the first method, unmodified, non-derivitized glycerin is utilized as solute, and the purpose is to quantitatively measure osmosis rates under the varying parameters of 1) solute concentration, 2) temperature, and 3) osmosis direction. The solute concentration is varied by adjusting the volume ratio of glycerin to water. The temperature is varied by placing the entire U-tube apparatus at room temperature, in a refrigerator, or in an oven. When the same glycerin-in-water solution is in one arm of the U-tube, the osmosis direction is opposite depending on whether water or undiluted glycerin is in the other arm. Owing to the symmetry of a U-tube, water can flow in either direction during osmosis, with the direction dictated by the feed and draw solutions.

In the second method, unmodified, non-derivitized dextran is utilized as solute, and the purpose is to compare experimental measurements of osmotic pressure to that predicted by theory. As osmosis occurs and water (feed solution) flows from its arm into the dextran solution (draw solution) arm, there will be an increasing height difference in liquid levels. When equilibrium is reached, the osmotic pressure and hydrostatic pressure equalize and the height difference in liquid levels ceases to change. Using well-known equations for both osmotic pressure and hydrostatic pressure, the liquid level height difference experimentally observed at equilibrium is compared to calculated values based on theory. The U-tube apparatus disclosed in this invention, together with the method described above, is the first of its kind capable of passively determining osmotic pressure and comparing it to calculations based on theory.

Both the foregoing general description and the following detailed descriptions are exemplary and explanatory only. The accompanying drawings are included to provide a further understanding of the invention. The drawings constitute part of this disclosure, illustrate several embodiments of the invention, and together with the descriptions, explain the principles of the invention.

BRIEF DESCRIPTION OF THE DRAWINGS

FIG. **1**A is a front view of an assembled U-tube apparatus, with an open lid with the narrow tube inserted on the left arm and a closed lid on the right arm.

FIG. **1**B is a perspective view of an assembled U-tube apparatus without lids on the arms.

FIG. **2**A is a perspective view of the center mount of the U-tube apparatus.

FIG. **2**B is a cross-sectional view of a semipermeable membrane inside the center mount without a humectant coating.

FIG. **2**C is a cross-sectional view of a semipermeable membrane inside the center mount with a humectant coating.

FIG. **2**D is an exploded view of the center mount.

FIG. **3**A illustrates an open lid for U-tube arm, which is a hollow cylinder with a narrow tube that can be reversibly inserted into the inner diameter of the hollow cylinder.

FIG. **3**B illustrates a closed lid for U-tube arm.

FIG. **4** illustrates chemicals as well as tools for measuring and dispensing liquids: 1) unmodified, non-derivitized glycerin, 2) pre-weighed, unmodified, non-derivitized dextran of known molecular weight, 3) food coloring dye, 4) disposable beaker(s) or disposable graduated cylinder(s), and 5) disposable pipet(s).

FIG. **5** illustrates a flow diagram of a method to conduct osmosis experiments using unmodified, non-derivitized glycerin as solute to measure osmotic rates with variable parameters of solute concentration, temperature, and osmosis direction.

FIG. **6** illustrates a flow diagram of a method to conduct osmosis experiments using unmodified, non-derivitized, high molecular weight dextran as solute to measure hydrostatic pressure at equilibrium, determine osmotic pressure at equilibrium, and to compare with calculated values based on theory.

FIG. **7** is a graph showing difference in liquid height levels, h (mm), as a function of time, t (hours), when a 6 mole/liter (6M) glycerin solution with blue dye was in one arm of U-tube with either water or pure glycerin in the other arm. The ratio h/t is defined as the osmotic rate. In the experiments, open lids were used without the liquid rising into the narrow tube, the U-tube arms were not completely filled with liquid, so that h was the difference in liquid column heights between the two arms as osmosis occurred. Using the same U-tube and the same 6M glycerin with dye solution in one arm, when pure glycerin replaced water in the other arm, osmosis occurred in the opposite direction.

FIG. **8** is a graph showing how osmotic rate (h/t) varied depending on glycerin concentration (3M or 6M glycerin) and whether osmosis occurred versus water or versus pure glycerin, for two similar semipermeable membranes in two different U-tube apparatuses when temperature was maintained at ~25° C. (room temperature). In the experiments, open lids were used without the liquid rising into the narrow tube, the U-tube arms were not completely filled with liquid, so that h was the difference in liquid column heights between the two arms as osmosis occurred.

FIG. **9** is a graph showing how osmotic rate (h/t) varied when osmosis occurred at room temperature (~25° C.) compared to refrigerator temperature (~4° C.) using the same glycerin concentration (6M) but for two different semipermeable membranes in two different U-tube apparatuses. Inset graph shows h/t for two experiments. In the experiments, open lids were used without the liquid rising into the narrow tube, the U-tube arms were not completely filled with liquid, so that h was the difference in liquid column heights between the two arms as osmosis occurred.

FIG. **10** is a graph showing difference in liquid height levels, h (mm), as a function of time, t (hours), during a long-term osmosis experiment to reach equilibrium with 0.16 mM native dextran (based on number-average molecular weight M_n~250,000 g/mole) as solute solution and water as solvent. Closed lids were used on both U-tube arms to prevent water evaporation.

FIG. **11** is a graph showing the rise in liquid height level in the narrow tube of the open lid as a function of time, t (minutes), under three different conditions 1) a 6M glycerin

solution with blue dye (draw solution) in one arm of U-tube and water (feed solution) in the other arm, experiment conducted at room temperature, 2) a 6M glycerin solution with blue dye in one arm of U-tube (feed solution) and pure glycerin (draw solution) in the other arm, experiment conducted at room temperature, and 3) a 6M glycerin solution with blue dye in one arm of U-tube (draw solution) and water (feed solution) in the other arm, experiment conducted in refrigerator. In all three experiments, an open lid with the narrow tube inserted was used on the draw solution arm, which was completely filled with liquid so that liquid was rising into the narrow tube as osmosis occurred. In this configuration, the rise in liquid height level in the narrow tube was measured on the order of minutes, rather than hours.

DETAILED DESCRIPTION OF THE INVENTION

This invention describes an osmosis kit comprising components to assemble a U-tube apparatus, chemicals, supplies, and two methods of using the kit. The drawings are for illustrative purposes, and embodiments illustrate the characteristics and functionality of the apparatus, chemicals, and two methods in which specific steps and sequence are not to be limiting in scope. The sequence may occur in a different order, so long as it still performs the goals of the invention. The illustrative drawings are not intended to be to scale or to be exhaustive of all possible sizes and dimensions, which may be adjusted to fit user needs.

In the embodiment **10** shown in FIGS. **1**A and **1**B, there are two curved arms **12**, one on the left and one on the right, connected to a center mount **20**. Both curved arms are optically clear hollow tubes that may be filled with liquid. The curved arms **12** are interchangeable so that either curved arm **12** may be used on either side of the center mount **20** with no difference in functionality nor performance. The length of the arms determine the overall height of the U-tube, and may be designated by the user. Length measuring scales **14** enable measurement of liquid column heights. The entire U-tube apparatus is securely placed on a stand **16**. Two different types of lids on the arms may be used, a closed lid **18** that tightly caps an arm, or an open lid **32** with a removable narrow tube that enables more rapid measurements of liquid level changes.

In the embodiment **20** shown in FIG. **2**A, the center mount **20** contains a semipermeable membrane **22** affixed between two washers **24**. Depending on the type of semipermeable membrane used, the membrane may be uncoated (FIG. **2**B) or coated with a humectant coating **26** (FIG. **2**C). Glycerin, provided in the osmosis kit, is itself a humectant and may be used as the coating. The washer-membrane-washer sandwich is affixed inside a protective tube **28**. Each end (left and right) of the protective tube **28** accommodates a removable, non-rigid, deformable sleeve **30** that allows one curved arm **12** to be inserted in a watertight manner. The sleeves **30** are thin-walled and cylindrical in shape. The definition of removable is that a curved arm **12** can be inserted into or removed from a sleeve **30**, and a sleeve **30** can be inserted into or removed from a center mount **20**, without damaging any of the components, so that all components can be reused. The definition of non-rigid is that the shape of the sleeve **30** can be changed using manual pressure without the use of any external tools. The definition of deformable is that the shape, including the wall thickness, of the sleeve **30** can be reversibly changed when pressure is applied or removed. FIG. **2**D illustrates an exploded view of the center mount **20**.

The assembled U-tube apparatus **10** is watertight over the long term so that water flows through only the semipermeable membrane **22** during osmosis but does not leak through any other opening. All components of the U-tube apparatus, curved arms **12**, length measuring scales **14**, center mount **20**, closed lid **18**, open lid **32**, and stand **16** are washable and reusable. This design provides a watertight connection, in contrast to NeoSci 30-1125 Osmosis Simulation Activity Model. To be watertight, this design does not require threaded components, in contrast to U.S. patent 2018/0280881. This design does not require an external press tool to create the seal, in contrast to U.S. Pat. No. 8,888,145. The U-tube can be assembled, disassembled, and reassembled using only manual manipulation with minimal assembly and a minimum number of parts, in contrast to the PASCO Diffusion-Osmosis Apparatus ME-6940 and the osmosis device described by Rodriguez-Duenas, et. al, (2022). Finally, when the assembled U-tube apparatus **10** is filled with liquid in both curved arms **12**, it is possible to disconnect only one curved arm **12** without disturbing the liquid in the other curved arm **12**. A center mount **20** with only one liquid-filled curved arm **12** connected via a sleeve **30** remains watertight.

The embodiment in FIG. **3**A illustrates an open lid **32**, which consists of a thick-walled hollow cylinder **34** with a narrow tube **36** that can be reversibly inserted into the inner diameter of the hollow cylinder **34**. The outer diameter of the thick-walled hollow cylinder **34** fits tightly into the opening of the U-tube arm **12**. The outer diameter of the narrow tube **36** fits tightly into the inner diameter of the hollow cylinder **34**. The inner diameter of the narrow tube **36** allows the long tip of a disposable pipet **50** to fit inside. When an open lid **32** with narrow tube **36** inserted is placed on top of a U-tube arm **12**, and the arm **12** is completely filled with liquid, as liquid level rises during osmosis, liquid is forced to travel into the narrow tube **36**. Forcing liquid into a smaller diameter narrow tube **36** compared to a larger diameter U-tube arm **12** allows faster measurement of osmotic rates. If slower measurements of osmotic rates are desired, the U-tube arms **12** are not completely filled with liquid during experiments, and the use of any lid is optional. The embodiment in FIG. **3**B illustrates a closed lid **18**, which tightly caps a U-tube arm **12**. A closed lid **18** may be required for long term experiments because it prevents water evaporation.

The embodiment **40** in FIG. **4** illustrates some of the other components provided in the kit. These components include the following chemicals: unmodified, non-derivitized glycerin **44**, unmodified, non-derivitized, pre-weighed dextran of known molecular weight **46**, and a food coloring dye **42**. Disposable (plastic) beaker(s) **48** and disposable (plastic) pipet(s) **50** are also provided to enable users to measure and dispense liquids. Although not illustrated, the kit contains an instruction manual which will describe the pertinent methods in embodiments **60** and **80**.

Two methods (**60** and **80**) of using the U-tube apparatus are disclosed in this invention, and experiments may be performed with either method using the same assembled U-tube apparatus. The first method **60** is for the purpose of measuring osmotic rates as three experimental parameters are varied: solute concentration, temperature, and osmosis direction, as illustrated in FIG. **5**.

As shown in FIG. **5**, after assembling the U-tube apparatus (step **62**), the first variable parameter is the glycerin solute concentration (step **64**). The user prepares a glycerin solution of desired concentration, i.e., glycerin-in-water solution, by selecting the proper volume ratio of glycerin to

water, and a table is provided (step **66**). The glycerin solution (draw solution) is placed in one arm of the U-tube while water (feed solution) is placed in the other arm. If color is desired, food coloring dye may be added to the glycerin solution, although osmosis occurs with glycerin only without any dye. For slower experiments (on the order of hours to overnight), the glycerin solution (draw solution) and water (feed solution) are placed to the same height in their respective arms with initial height difference being zero, and arms are not completely filled with liquid to leave enough room for liquid to rise during osmosis (step **68**). For faster experiments (on the order of minutes to hours), the arm with glycerin solution (draw solution) has an open lid with narrow tube inserted, and is completely filled, so that during osmosis, liquid rises in the narrow tube (step **68**). The rise in liquid level in a smaller diameter narrow tube **36** will be significantly faster than the rise in liquid level in a larger diameter U-tube arm **12**.

The second variable parameter is temperature, and an experiment is conducted at room temperature (step **70**), in a refrigerator above freezing point of water, or in an oven below boiling point of water (step **72**). Whether at room temperature, in a refrigerator, or in an oven, osmosis occurs with water (feed solution) flowing from its arm into the glycerin solution (draw solution) arm, and over time, the liquid level height in the water arm decreases while the liquid level height in the glycerin solution arm increases. The osmotic rate may be determined by the difference in liquid level heights (h) between the arms as a function of time (t), or by the rise in liquid level in the narrow tube when it is used.

The third variable parameter is osmosis direction, and successive experiments can be conducted without disassembling the U-tube. If user desires osmosis to occur in the same direction in U-tube (step **74**), then water (feed solution) remains in its arm, while a glycerin-in-water solution (draw solution) is replaced with a glycerin-in-water solution of different concentration, and process is repeated. It is possible to remove liquids from U-tube arms using a pipet **50** provided in the kit. It is also possible to disassemble and reassemble only one arm of U-tube without disturbing the liquid in the other arm, because each U-tube arm **12** remains watertight once inserted into the center mount **20** via a sleeve **30**.

If user desires osmosis to occur in the opposite direction in U-tube (step **76**), then the glycerin-in-water solution remains in its arm, and water is replaced with pure glycerin in the other arm. In this case, the glycerin-in-water solution becomes the feed solution and pure glycerin becomes the draw solution, and the direction of osmosis reverses with water (solvent) flowing from the glycerin-in-water arm into the pure glycerin arm.

FIG. **6** illustrates another method embodiment **80** of using the osmosis kit, for the purpose of experimentally determining the osmotic pressure at equilibrium and comparing it to theoretical prediction. After assembling a U-tube apparatus per instructions manual (step **82**), an unmodified dextran solute solution of pre-determined concentration is prepared by dissolving pre-weighed dextran powder **46** in a known amount of water (step **84**). The unmodified dextran solution (draw solution) is placed into one arm of the U-tube and water (feed solution) is filled to the same height in the other arm with initial height difference being zero (step **86**). This method involves a long-term experiment to reach equilibrium, and closed caps **18** may be used to prevent water evaporation. As osmosis occurs, water flows from its arm into the dextran solution arm, resulting in a liquid level height difference that increases with time. The higher liquid level in the dextran solution arm exerts a hydrostatic pressure, due to the force of gravity, that interferes with osmosis. Eventually, when equilibrium is reached, the height difference in liquid levels ceases to change because osmotic pressure and hydrostatic pressure equalize. Water flow in is the same as water flow out.

At equilibrium, the height difference observed experimentally in the U-tube can be compared to theoretical prediction by setting the osmotic pressure equal to the hydrostatic pressure (step **88**), and both pressures are described by well-established equations (step **88**). A table of unmodified dextran solution density (step **90**) is provided because determination of a solution's hydrostatic pressure requires its density. Another table is provided with calculated theoretical values of liquid level height differences expected at equilibrium for a range of unmodified dextran concentrations (step **92**).

The kit and methods described herein may be better understood with reference to the accompanying examples, which are intended for illustrating but not limiting the invention.

EXAMPLE 1

Osmosis experiments were performed using a U-tube apparatus **10** (FIGS. **1**A and **1**B) with open lids **32** (FIG. **3**A) on both arms, following method **60** (FIG. **5**). A solution of 6M glycerin with blue dye (0.2 mM) was placed in right arm of U-tube while water was placed in left arm to the same height level (initial height difference in liquid level between the two arms was zero, i.e., h=0 at t=0). Arms were not completely filled with liquid and height difference in liquid columns between the two arms (h) was measured as a function of time (t). Osmosis occurred with water flowing from its arm across the semipermeable membrane into the 6M glycerin with blue dye arm, from left to right. Afterwards, water was pipetted out of its arm and replaced with pure (i.e., undiluted) glycerin, with the same 6M glycerin and dye solution remaining in its arm. Osmosis occurred with water flowing from the 6M glycerin and dye solution arm across the semipermeable membrane into the pure glycerin arm, in the opposite direction, from right to left. Shown in FIG. **7** are experimental data observed for 6M glycerin with dye vs. water and 6M glycerin with dye vs. pure glycerin. In both cases, linear regression of h/t showed excellent linear fit (goodness-of-fit measure R^2>0.99). The ability to remove liquid (e.g., water) from its arm and replace with another liquid (e.g., glycerin) without disassembling the U-tube apparatus reduced time between experiments.

EXAMPLE 2

In this example, method embodiment **60** (FIG. **5**) was used with solute concentration (steps **64** and **66**) and osmosis direction (steps **74** and **76**) as the variable parameters. Two U-tube apparatuses were utilized with semipermeable membranes made of similar, but not identical, materials used for dialysis. Open lids on both arms were used in all experiments, and both arms were filled to the same liquid level, but not completely full, and height difference in liquid columns between the two arms (h) was measured as a function of time (t) with h=0 at t=0 in each experiment. Four experiments were performed in each apparatus at room temperature back-to-back without disassembling apparatus between experiments: 1) 3M glycerin with blue dye vs.

water, 2) 3M glycerin with blue dye vs. pure glycerin, 3) 6M glycerin with blue dye vs. water, and 4) 6M glycerin with blue dye vs. pure glycerin. FIG. **8** shows the results of osmotic rate h/t for both U-tube apparatuses with 3M glycerin and 6M glycerin, vs. water and vs. pure glycerin. In each case, whether 3M or 6M glycerin was in a U-tube arm, when water was replaced with pure glycerin in the other arm, osmosis occurred in the opposite direction. Osmotic rate h/t was linear in all experiments with goodness-of-fit measure $R^2>0.99$. As observed in FIG. **8**, results were similar between the two U-tubes since both had similar membrane material.

EXAMPLE 3

In this example, method embodiment **60** (FIG. **5**) was used with temperature (steps **70** and **72**) as the variable parameter. Two U-tube apparatuses were used with semipermeable membranes different from each other and different from those used in EXAMPLES 1 and 2. One apparatus contained a semipermeable membrane with smaller pores, while the other apparatus contained a semipermeable membrane with larger pores. Similar to EXAMPLES 1 and 2, open lids were used on both arms, arms were not completely filled with liquid, and height difference in liquid columns between the two arms (h) was measured as a function of time (t) with h=0 at t=0. As expected, osmotic rate (h/t) was faster when pores in the membrane were larger, and h/t was faster at higher temperature (FIG. **9**). This example demonstrates that it is possible to obtain faster osmotic rates by changing the membrane. Also seen in FIG. **9** is that for both U-tubes, when apparatus was placed in refrigerator, h/t was slower at ~4° C. than that at room temperature (~25° C.), confirming that temperature does indeed affect osmosis. As observed in EXAMPLES 1 and 2, the osmotic rate h/t was linear in all experiments with goodness-of-fit $R^2>0.99$ (FIG. **9**).

Data collected in EXAMPLES 1 to 3 with goodness-of-fit $R^2>0.99$ were possible because liquid glycerin was used as solute for osmosis. The center mount was reused in multiple experiments, saving time as well as cost. In the present disclosure, if the semipermeable membrane, e.g., dialysis membrane, needs to be maintained wet, glycerin itself is a humectant, and can be applied as a surface coating to the membrane. The present invention is not limited to dialysis membrane, which is soft and pliable, but permits any semipermeable membrane to be sandwiched between washers in the center mount.

EXAMPLE 4

Using method embodiment **80** (FIG. **6**), experimental data for osmotic pressure was compared to calculated theoretical prediction. In this example, the experiment was long term (>45 days) and closed lids were placed on both arms of U-tube. High molecular weight unmodified dextran, not glycerin, was utilized as solute because the purpose of the experiment was to determine osmotic pressure, not osmotic rate. Dextran is a water soluble, biocompatible, and nontoxic polysaccharide. The molecular weight of dextran may vary from thousands to millions grams/mole (g/mol), and is an average value within a distribution of values because a precisely controlled molecular weight is nearly impossible. The reported average may be a number averaged molecular weight (M_n) or a weight averaged molecular weight (M_w), with M_w greater than M_n. The polydispersity index (M_w/M_n) is a measure of the broadness of the molecular weight distribution. For relatively high molecular weight dextran, a polydispersity index of 2 (M_w/M_n~2) is typical.

In this example, unmodified dextran with M_w~500,000 g/mol was selected for osmotic pressure testing. In order to compare experimental results with theoretical prediction, the solute solution (draw solution) must be very dilute, and therefore the solute must have high molecular weight, e.g., in the range of 10^5 to 10^6 g/mol. Glycerin is not suitable for this purpose because its molecular weight is only 92 g/mol. A 0.08 mM unmodified dextran solution based on M_w of 500,000 g/mol, same as a 0.16 mM unmodified dextran solution based on M_n of 250,000 g/mol, was placed in one arm of U-tube while water was placed in the other arm, with h=0 at t=0. When equilibrium is reached, the liquid level heights no longer change. In this example, the observed h in the U-tube was compared to what is predicted by theory using tables **90** and **92** (FIG. **6**). As seen in FIG. **10**, h was approaching ~40 mm after 1100 hours and beginning to plateau. A value of about 40 mm for h is what would be expected for a solute solution of 0.16 mM concentration (table **92**, FIG. **6**). This example demonstrates that with a properly configured U-tube and an appropriate method, it is possible to directly compare experimental results with theoretical predictions.

EXAMPLE 5

In this example, method embodiment **60** (FIG. **5**) was used with temperature (steps **70** and **72**) and osmosis direction (steps **74** and **76**) as the variable parameters. The difference between this example (EXAMPLE 5) and the previous examples (EXAMPLES 1 to 3) is that liquid in the draw solution arm was forced into the narrow tube **36** inserted in an open lid **32**. The draw solution arm was completely filled with liquid, and as osmosis occurred, liquid traveled up the narrow tube **36**, and osmotic rate was determined by the rise in liquid level in the narrow tube (mm) as a function of time. In this case, a steady rise in liquid level in the narrow tube **36** could be measured within the order of minutes, instead of hours as in previous examples. Similar to EXAMPLES 1 and 2, with the same 6M with blue dye solution in one arm of the U-tube, when pure glycerin replaced water in the other arm, osmosis occurred in the opposite direction and h/t was slower. Similar to EXAMPLE 3, when osmosis occurred vs. water and the apparatus was placed in the refrigerator, h/t was slower at ~4° (refrigerator) compared to ~25° C. (room temperature). This example, however, shows that much more rapid measurements of osmotic rates are possible by measuring the rise in liquid level in a narrow tube **36** instead of measuring the difference in liquid height levels between two U-tube arms **12**.

The invention claimed is:

1. A method for determining osmotic rate, or the rate of osmosis, using a U-tube apparatus comprising a U-tube having two arms separated by a forward osmosis membrane, comprising steps of:

(a) preparing solutions of unmodified glycerin-in-water with known glycerin concentration by adjusting volume ratio of glycerin to water;

wherein a table is provided listing volume of unmodified glycerin and volume of water for varying concentrations of glycerin-in-water solutions;

(b) filling one arm of the U-tube with water as a feed solution and filling the other arm of the U-tube with

glycerin-in-water as the draw solution for osmosis to occur with water flowing from its arm into the glycerin-in-water arm;

(c) with same glycerin-in-water solution in its arm of the U-tube, replacing water with pure or undiluted glycerin in the other arm of the U-tube for osmosis to occur in the opposite direction with water flowing from the glycerin-in-water arm into the pure or undiluted glycerin arm;

(d) performing slower osmosis experiments wherein osmosis is allowed to occur for more than 180 minutes, filling both arms of the U-tube to same liquid level height and measuring the difference in liquid level heights between the two arms as a function of time;

(e) performing faster osmosis experiment wherein osmosis is allowed to occur up to 180 minutes, using an open lid with narrow tube inserted in draw solution arm of the U-tube inserted, filling draw solution arm of the U-tube completely and allowing liquid to enter the narrow tube as osmosis occurs, and measuring the rise in liquid level inside the narrow tube as a function of time;

(f) performing the steps (d) and (e) at room temperature, inside a refrigerator, or inside an oven wherein osmosis occurs at different temperatures;

(g) recording and plotting the difference in liquid level heights between the two arms of the U-tube as a function of time to determine the osmotic rate as performed in step (d) and recording and plotting the rise in liquid level height inside the narrow tube as a function of time to determine the osmotic rate as performed in step (e).

* * * * *
